# An optimized 16S–23S rRNA intergenic spacer region PCR for the detection and identification of *Bartonella* spp.

**DOI:** 10.1128/spectrum.02964-25

**Published:** 2026-02-04

**Authors:** Alexander A. Dichter, Rebecca Kaufmann, Luis Solis Cayo, Pablo Tsukayama, Volkhard A. J. Kempf

**Affiliations:** 1 Institute for Medical Microbiology and Infection Control, University Hospital, Goethe University 72118, Frankfurt am Main, Germany; 2 Laboratorio de Microbiología Molecular y Biotecnología, Facultad de Ciencias Biológicas, Universidad Nacional Mayor de San Marcos 569249 , Lima, Peru; 3 Laboratorio de Genómica Microbiana, Facultad de Ciencias e Ingeniería/Instituto de Medicina Tropical “Alexander von Humboldt”, Universidad Peruana Cayetano Heredia 504779 , Lima, Peru; University of Heidelberg, Heidelberg, Germany

**Keywords:** *Bartonella *spp., neglected tropical disease, molecular diagnostics, emerging pathogens

## LETTER

**Fig 1:**
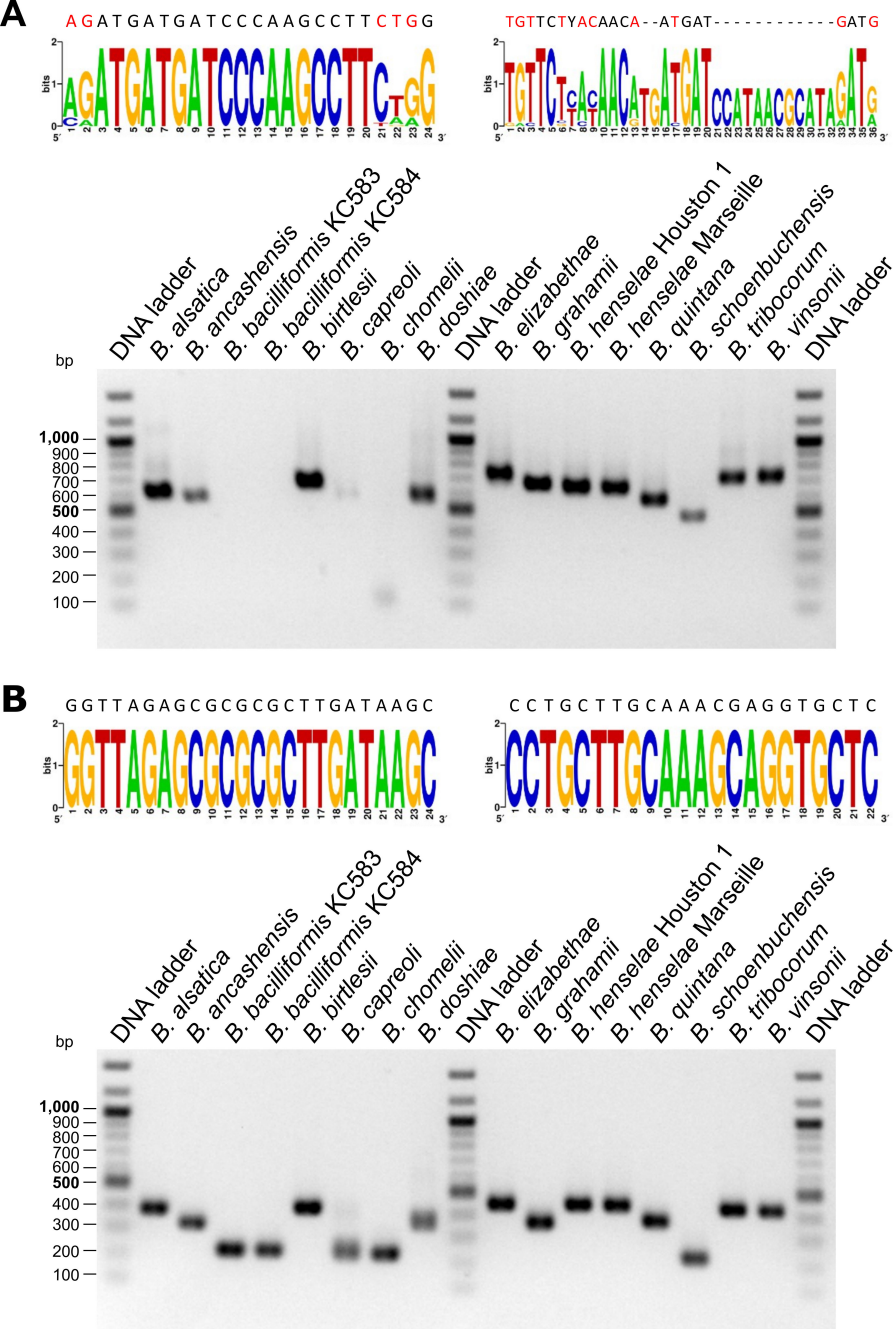
Primer binding sites and PCR amplification results (agarose gel electrophoresis) targeting the *Bartonella* ITS region. (**A**) Previously published primers (6) and (**B**) primers designed based on the consensus sequence established in this study were used. The consensus sequence of the ITS region and the corresponding primer sequences are shown in the subfigures, with mismatches indicated in red.

The emerging genus *Bartonella* comprises 60 species with unknown medical relevance (National Center for Biotechnology Information, Taxonomy Browser, 5 December 2025 [1]). Some species are human pathogenic (e.g., *B. henselae*: cat scratch disease, bacillary angiomatosis; *B. quintana*: trench fever, endocarditis; and *B. bacilliformis*: Carrion’s disease); other species (e.g., *B. alsatica*: endocarditis and *B. grahamii:* lymphadenopathy) are only rarely described to cause human infections (2). Accurate diagnosis of *Bartonella* infections remains challenging due to unspecific clinical manifestations, difficulties in cultivation, genetic diversity within the genus, and frequent occurrence of low-level bacteremia in infected patients (3). Therefore, because of the fact that serological diagnostics have been developed only for *B. henselae*, *B. quintana* (4), and recently for *B. bacilliformis* (5), PCR-based assays have become indispensable for pathogen detection and differentiation. Pioneer work of Maggi and Breitschwerdt established the 16S–23S rRNA intergenic spacer (ITS) region as a valuable diagnostic target widely used in molecular diagnostics of *Bartonella* infections. This region is highly variable in sequence and length between *Bartonella* species and is embedded between conserved flanking regions, allowing the design of universal primers and enabling a reliable discrimination among closely related *Bartonella* species (6). Despite this great contribution to diagnostics, our results revealed that this PCR protocol with the described ITS primers fails to amplify particular species that were later fully sequenced or discovered because of numerous mismatches to the ITS target sequence (Fig. 1A). This limitation might lead to false-negative results in both clinical and epidemiological studies, ultimately impacting patient management or public health surveillance. Here, we aimed to optimize an ITS-PCR (targeting a sequence of the 16S–23S rRNA ITS) for improved diagnosis of clinically relevant *Bartonella* species.

Genomic sequences from 16 *Bartonella* species (Table 1) were aligned using MAFFT v.7.490 (Geneious, Dotmatics, Auckland, New Zealand). Only 16S–23S rRNA ITS sites that were 100% conserved across all aligned sequences were considered for primer construction, covering the major pathogenic and novel emerging species (BsppITS-386s: 5′-GGTTAGAGCGCGCGCTTGATAAGC-3′ [3′ end of the 16S rRNA gene] and BsppITS-824as: 5′-CCTGCTTGCAAAGCAGGTGCTC-3′ [5′ end of the 23S rRNA gene]) generating PCR amplicons between 207 and 438 nucleotides.

**TABLE 1:** *Bartonella* strains used in this study for the development of an optimized 16S–23S intergenic spacer region PCR

*Bartonella* strains	Accession no. of reference genome^*a*^	PCR amplicon size
*B. alsatica* IBS 382 (CIP105477)	CP058235	367
*B. ancashensis* A07	CP180576	311
*B. bacilliformis* KC583	CP000524	255
*B. bacilliformis* KC584	CP045671	255
*B. birtlesii* CIP106294	KE007210	407
*B. capreoli* CIP 106691	CADDZX010000017	207
*B. chomelii* A828	NZ_JACJIR010000013	219
*B. doshiae* ATCC 700133	CACVBE010000020	329
*B. elizabethae* ATCC 49927	NZ_LR134527	438
*B. grahamii* ATCC 700132	CP001562	352
*B. henselae* Houston 1	NZ_CP072903	438
*B. henselae* Marseille	NZ_CP072904	438
*B. quintana* JK31	KL411732	367
*B. schoenbuchensis* DSM 13525	NZ_CP154603	207
*B. tribocorum* CIP10476	AM260525	424
*B. vinsonii* ATCC 51672	CADEAK010000021	406

^*a*^ Reference genomes were taken from https://www.ncbi.nlm.nih.gov.

DNA was extracted from *Bartonella* cultures and 12 bacterial control strains (Table S1). PCR was performed under certified conditions (DIN EN ISO 15189:2024, certificate number D-ML-13102-01-00): initial denaturation (95°C/3 min), followed by 35 cycles (denaturation 94°C/20 s; annealing: 60°C/30 s, elongation: 72°C/40 s, final extension 72°C/1 min). Products were analyzed by agarose gel electrophoresis, demonstrating a robust amplification across all tested strains, including *B. bacilliformis* and *B. ancashensis* (Fig. 1B).

**Fig 2:**
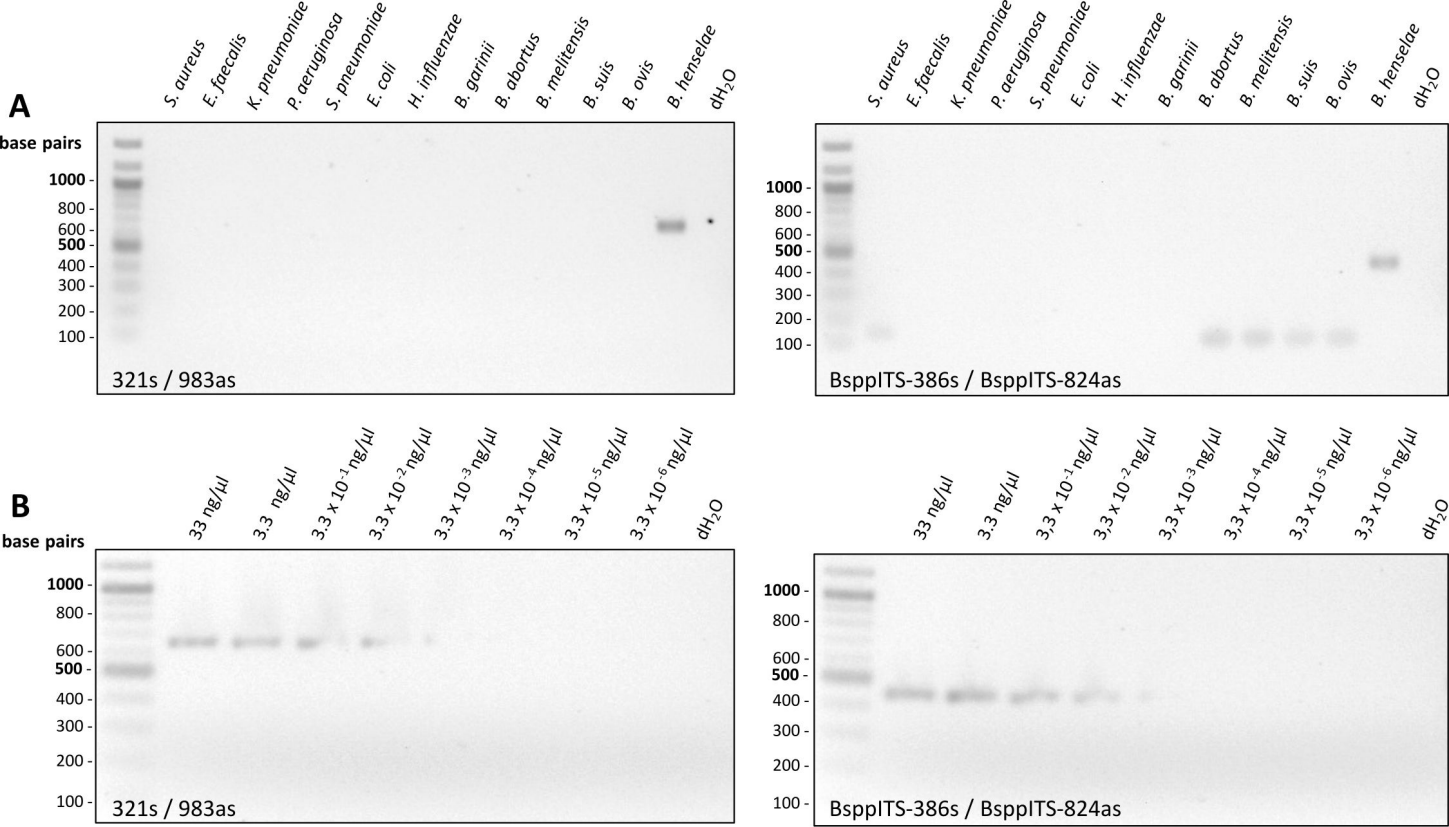
Determination of (**A**) specificity and (**B**) sensitivity of the 16S–23S rRNA intergenic spacer region PCRs. (**A**) Control bacteria (see Table S1) were grown under standard laboratory conditions. Positive control: *B. henselae* Marseille, negative control: *aqua dest*. (dH_2_O). (**B**) Sterile human blood samples were spiked with DNA from *B. henselae* Marseille at the indicated final concentrations (ranging from 33 to 3.3 × 10^−6^ ng/µL). Negative control: *aqua dest*. (dH_2_O). Left panels: Primers 321s/983as (described in reference 6). Right panels: Primers BsppITS-386s/BsppITS-824as (described in this study).

*In silico*, PCR products were aligned using MAFFT to assess sequence variability within the amplified ITS region to evaluate the potential for species-level discrimination. This *in silico* sequence analysis of the expected amplicons revealed unique DNA sequences except for *B. capreoli* and *B. schoenbuchensis* (Table S2), confirmed by Sanger sequencing (data not shown). Specificities of the old and new PCR protocols were evaluated using a broad selection of 12 clinically relevant bacterial strains (Fig. 2A; Table S1), demonstrating no unspecific amplicons between 207 and 438 bp, not even for the four closely related *Brucella* spp. PCR sensitivities were evaluated by spiking sterile human blood samples with DNA from *B. henselae* Marseille (final concentrations: 33 ng/µL to 3.3 × 10^−6^ ng/µL). DNA was extracted using the QIAsymphony DSP Virus/Pathogen Kit (Qiagen, Hilden, Germany) under certified conditions (DIN EN ISO 15189:2024), and the 16S–23S rRNA ITS region was amplified using both the old and new PCR protocol. Data revealed that both PCRs have a comparable sensitivity (3.3 × 10^−3^ ng/µL, Fig. 2B). As the 16S–23S rRNA ITS region is present twice in the genomes of *Bartonella* spp., it might represent a PCR target resulting in more sensitive PCRs compared to PCR targets with only one copy (16S-rDNA, *ftsZ*, *gltA*, *groEL*, *ribC*, and *rpoB*). This speculation, however, has not been addressed experimentally.

Our study underscores the importance of periodically re-evaluating diagnostic primers in PCR-based diagnostic approaches as new bacterial species are identified. In regions where *Bartonella*-related illnesses are endemic, implementation of the optimized ITS primers could improve diagnostic accuracy, facilitate timely treatment, and support more reliable molecular epidemiology.

## Data Availability

All data are provided within the article and the supplemental material.

## SUPPLEMENTAL MATERIAL

Supplemental tables spectrum.02964-25-s0001.pdf Tables S1 and S2.

ASM does not own the copyrights to Supplemental Material that may be linked to, or accessed through, an article. The authors have granted ASM a non-exclusive, world-wide license to publish the Supplemental Material files. Please contact the corresponding author directly for reuse.
