# Supplementary material for: An optimized 16S–23S rRNA intergenic spacer region PCR for the detection and identification of Bartonella spp
Source: Microbiol Spectr. 2026 Feb 4;14(3):e02964-25. doi: 10.1128/spectrum.02964-25 (PMC12955397; doi:10.1128/spectrum.02964-25)
Supplement: Supplemental tables — Tables S1 and S2. [file spectrum.02964-25-s0001.pdf]

**Supplementary Table 1.** Bacteria used for specificity evaluation of the optimized 16S-23S-ITS region PCR.

| ***bacterial strains*** | **reference or laboratory strain designation** |
|---|---|
| *Brucella abortus* | patient isolate KB1-2023-03-27-01 |
| *Brucella mellitensis* | patient isolate KB1-2023-03-27-02 |
| *Brucella suis* | patient isolate KB1-2023-03-27-03 |
| *Brucella ovis* | patient isolate KB1-2023-03-31-01 |
| *Borrelia garinii* subsp. *bavariensis* (*) | Pbi / ATCC BAA-2496 |
| *Enterococcus faecalis* | ATCC 29212 |
| *Escherichia coli* | ATCC 25922 |
| *Haemophilus influenzae* | ATCC49766 |
| *Klebsiella quasipneumoniae* | ATCC 700603 |
| *Pseudomonas aeruginosa* | ATCC 27853 |
| *Staphylococcus aureus* | ATCC 25923 |
| *Streptococcus pneumoniae* | ATCC 49619 |

* Hördt A, López MG, Meier-Kolthoff JP, Schleuning M, Weinhold LM, Tindall BJ, Gronow S, Kyrpides NC, Woyke T, Göker M. 2020. Analysis of 1,000+ Type-Strain Genomes Substantially Improves Taxonomic Classification of *Alphaproteobacteria*. *Front Microbiol* 11:468. doi: 10.3389/fmicb.2020.00468.

**Supplementary Table 2.** Percent identity of ITS PCR amplicons (*in silico* analysis).

| | | 1 | 2 | 3 | 4 | 5 | 6 | 7 | 8 | 9 | 10 | 11 | 12 | 13 | 14 | 15 | 16 |
|---|---|---|---|---|---|---|---|---|---|---|---|---|---|---|---|---|---|
| ***B. alsatica*** | **1** | 100 | | | | | | | | | | | | | | | |
| ***B. ancashensis* A07** | **2** | 43.77 | 100 | | | | | | | | | | | | | | |
| ***B. bacilliformis* KC583** | **3** | 39.42 | 42.6 | 100 | | | | | | | | | | | | | |
| ***B. bacilliformis* KC584** | **4** | 39.42 | 42.6 | 100 | 100 | | | | | | | | | | | | |
| ***B. birtlesii*** | **5** | 59.57 | 41.43 | 33.65 | 33.65 | 100 | | | | | | | | | | | |
| ***B. capreoli*** | **6** | 39.57 | 40.31 | 65.78 | 65.78 | 33.57 | 100 | | | | | | | | | | |
| ***B. chomelii*** | **7** | 40.64 | 38.81 | 62.82 | 62.82 | 34.78 | 93.61 | 100 | | | | | | | | | |
| ***B. doshiae*** | **8** | 62.2 | 45.68 | 41.88 | 41.88 | 55.53 | 40.17 | 41.62 | 100 | | | | | | | | |
| ***B. elizabethae*** | **9** | 46.84 | 37.97 | 34 | 34 | 47.07 | 34.77 | 35.91 | 41.39 | 100 | | | | | | | |
| ***B. grahamii*** | **10** | 46.67 | 42.6 | 42.66 | 42.66 | 43.81 | 42.66 | 43.26 | 44.11 | 69.63 | 100 | | | | | | |
| ***B. henselae* Houston 1** | **11** | 50.34 | 41 | 34.99 | 34.99 | 50.88 | 34.02 | 35.16 | 46.98 | 49.59 | 46.17 | 100 | | | | | |
| ***B. henselae* Marseille** | **12** | 50.34 | 41 | 34.99 | 34.99 | 50.88 | 34.02 | 35.16 | 46.98 | 49.59 | 46.17 | 100 | 100 | | | | |
| ***B. quintana* JK 31** | **13** | 46.05 | 39.86 | 37.99 | 37.99 | 52.57 | 41.44 | 42.78 | 50.74 | 45.42 | 40.18 | 47.32 | 47.32 | 100 | | | |
| ***B. schoenbuchensis*** | **14** | 39.57 | 40.31 | 65.78 | 65.78 | 33.57 | 100 | 93.61 | 40.17 | 34.77 | 42.66 | 34.02 | 34.02 | 41.44 | 100 | | |
| ***B. tribocorum*** | **15** | 45.18 | 38.78 | 35.57 | 35.57 | 46.3 | 35.68 | 36.21 | 41.41 | 79.27 | 70.96 | 47.69 | 47.69 | 46 | 35.68 | 100 | |
| ***B. vinsonii* subsp. *berkhoffii*** | **16** | 40.09 | 38.69 | 30.99 | 30.99 | 47.73 | 35.8 | 34.57 | 39.36 | 45.09 | 43.46 | 44.3 | 44.3 | 41.23 | 35.8 | 45.01 | 100 |
